# Masticatory Function and Oral Health-Related Quality of Life After Immediate Prosthetic Therapy for Geriatric In-Hospital Patients: A Retrospective Cohort Study

**DOI:** 10.3390/dj14050268

**Published:** 2026-05-04

**Authors:** Michael Pampel, Jana Kraft, Johannes W. Kraft

**Affiliations:** 1 Private Practice, TMD-Center, Sana Hospital, 96450 Coburg, Germany; 2 University Postgraduate Study Program “Evidence Based Medicine”, School of Medicine, University of Split, 21000 Split, Croatia; 3 Faculty of Social Work and Health, University of Applied Sciences, 96450 Coburg, Germany; jkraft-laurentius@web.de (J.K.);; 4 Rehabilitation Department, Sana Hospital, 96450 Coburg, Germany

**Keywords:** geriatrics, intra-oral risk factors, masticatory function improvement, oral health-related quality of life, treatment satisfaction

## Abstract

**Objectives**: The impact of dental and oral health conditions on the oral health state and oral health-related quality of life (OHRQL) in geriatric in-hospital (GIH) patients requires clarification. For this reason, this exploratory and hypothesis-generating study aimed to examine the associations between intra-oral risk factors (IRFs), masticatory functions (MFs), and treatment satisfaction (TS) in this context. **Methods**: This retrospective cohort study enrolled a total of 81 patients requiring immediate prosthetic treatment (IPT) by consultant dentists at Sana Hospital, Coburg, Germany. The evaluation focused on oral and dental state and MFs such as a modified DMFT and Eichner Index (EI), as well as on IRFs such as inflammation, mucogingival junction insufficiency (MGJI), severe bone crest atrophy (SBCA), and oral pain. The outcomes were measured after IPT using MF improvement (MFI) as the primary outcome and TS as the secondary outcome. **Results**: The outcomes (*p* < 0.05) were evaluated using objective measures such as the calculations of the EI, the EI modifications and the modified DMFT index. The results were associated, respectively, with the different IRFs and with specific SBCA. A total of 85% of GIH patients suffered from severe bone crest atrophy (SBCA), which showed a significant association with MF measured using EI after IPT (*p* = 0.001). MFI after IPT was associated with the EI before IPT (*p* < 0.000). The achieved MFI was finally associated with the untreated sample (all patients, *p* < 0.000) and with the presence or absence of the following IRFs at baseline: inflammation (*p* < 0.003), absence of oral pain (*p* < 0.000), presence of SBCA (*p* < 0.000), and absence of MFI (*p* < 0.000). A total of 78% of GIH patients confirmed treatment satisfaction (TS) after IPT, which was significantly and strongly associated with SBCA (*p* < 0.003). **Conclusions**: Oral health was primarily impaired by SBCA and can be measured by the analysis of EI and DMFT associations, which provided MFI and TS as outcomes for OHRQL.

## 1. Introduction

The World Dental Federation (FDI) confirmed in 2015 that oral health is closely related to general health and people’s quality of life through its influence on oral functions and oral health-related quality of life (OHRQL) [1]. Previous research on individual components of OHRQL revealed the importance of dental and oral factors, among others, regarding OHRQL [2]. The significant association between edentulism and OHRQL was confirmed recently by measuring the oral impact on daily performance (ODIP) [3]. Complete and partial edentulism are significant reasons for reduced OHRQL. Furthermore, the need for dental treatment is growing due to growing life expectancy and demographic transformation. This was recently emphasized because the World Health Organization (WHO) estimated that the proportion of people over the age of 60 globally will almost double from the current 12–22% by 2050 [4]. Originally, in 2003, the WHO called for continuous improvement in oral health during the 21st century, reaffirming this goal in 2018 by focusing on promoting oral health and quality of life among older adults [5,6]. OHRQL consists of objective and subjective items.

The treatment rules of the WHO recommend no fewer than 20 natural teeth. Additionally, at least four occlusal units of posterior and anterior teeth are required to maintain chewing as a key daily activity of life [5,6]. OHRQL consists of objective and subjective items. Therefore, it is not directly and completely detectable, but assessable by questionnaires [7,8,9]. There are the basic conditions for good OHRQL and depend on healthy mastication through healthy teeth or sufficient dentures. Masticatory functions can be assessed using index calculations such as the EI and DMFT indices. These indices represent masticatory functions and have been confirmed as objective and practicable measures through clinical examinations of elderly patients [10]. The association between the number of teeth and OHRQL has been confirmed in contemporary studies [4,11]. A previous study confirmed that mastication and OHRQL can be improved after treatment with new complete denture incorporation [12]. Existing comprehensive outcome measures are expensive [13,14,15] and unaffordable, and there is no practical bedside method available for the objective evaluation of patients’ masticatory function.

The concrete effects of oral conditions on quality of life can be assumed as follows. Good mastication is the best precondition for good digestion and healthy metabolism, with a positive impact on physical and mental wellbeing. Participation in public and private activities of daily life is an integral part of quality of life. In the context of oral health-related quality of life, sufficient masticatory function with natural teeth or well-fitting and functional dentures is the most important cause of TS and OHRQL, specifically in older people and those with reduced general health.

In this study, we evaluated the relationships among intra-oral risk factors and focused on index calculations as the most relevant measure of masticatory function. Therefore, we used masticatory function improvement (MFI) as a clinical measure of the Eichner and DMFT index in this study protocol [16,17] because it best reflects the objective aspects of mastication for in-hospital assessment of OHRQL in GIH patients. In cases of disorders in the oral cavity or impaired prosthodontic function, GIH patients required urgent care through immediate prosthetic treatment (IPT). The aim of the study was to research whether there are associations between oral status, mastication, and treatment satisfaction. The hypothesis was that there would be an association between these factors and the partial evidence.

## 2. Methods

### 2.1. Participants, Sampling Methods, Study Design and Examiners

**Figure 1:**
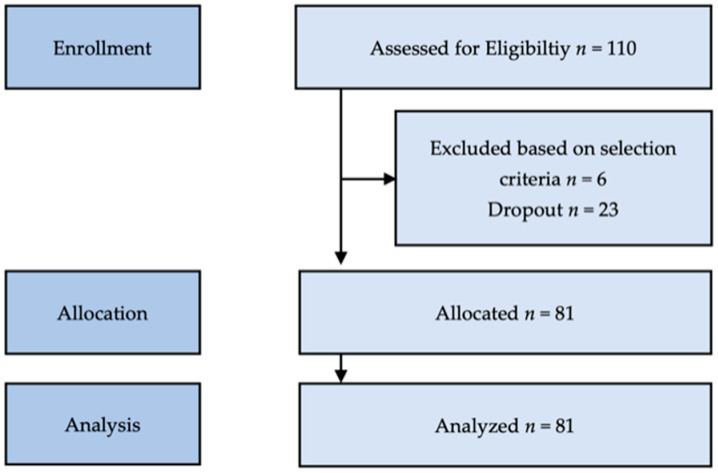
Flowchart depicting the patient selection process.

A total of 110 patients were consecutively recruited from the Rehabilitation Department at Sana Hospital in Coburg between 1 January 2015 and 30 June 2017 (see flowchart in Figure 1). All patients signed an informed consent form independently and in person after individual explanation and agreement. The study population consisted of individuals aged >60 years who were hospitalized for rehabilitation and required immediate dental intervention due to signs of malnutrition, pathological findings, or complaints related to the oral cavity, such as pain, reduced chewing capacity, soft tissue inflammation, lack of teeth, or insufficient dentures. Preliminary patient screening was conducted by physicians according to predefined inclusion criteria. Prior to dental treatment, baseline data on oral, dental, and functional status was collected, marking the onset of the study. Patients with conditions directly affecting masticatory functions, including palliative-stage cancer of the digestive organs or head and neck, as well as those who had undergone surgery in these regions, were excluded from the analysis (*n* = 6). An additional 23 patients were excluded due to dropout and incomplete datasets. The final study cohort comprised 81 participants. A priori power analysis was conducted to determine the required sample size for a two-tailed paired *t*-test comparing dependent means using G*Power (version 3.1.94). Assuming a small-to-moderate effect size (*d*_x_ = 0.3), an alpha level of 0.05, and statistical power of 0.80, the analysis yielded a required sample size of 90 participants. The study was designed and executed as a retrospective cohort investigation [18]. The examiners consisted of the principal investigator (MP) and one assistant dentist per year, who were duly and legally qualified dentists. They were introduced and calibrated in the specific evaluation. Because of their small contribution, which was based on simple dental assessment and the use of standard index calculation, the Kappa coefficients can be assumed as 0.6–0.8 for inter-examiner reliability. Dental assessment and treatment were conducted in the hospital’s dental office.

### 2.2. Cohort Characterization and Prosthetic Treatment Specification

This study cohort suffered from very high general and dental morbidity, but the majority of patients were willing and able to undergo different kinds of rehabilitation treatment and demonstrated relevant cooperation and effort depending on their actual status and cognition. Most individuals wished to be discharged from the rehab department to live at home, either independently or with home care, while maintaining satisfactory participation in daily life activities for as long as possible. For this very vulnerable population, the treatment involved the fastest possible prosthetic care from diagnosis to incorporation. This was implemented based on the severity of the patient’s health state, with a scheduled hospitalization of three weeks [18]. Functional rehabilitation of mastication was completed within one to three days from diagnosis to the incorporation of repaired or relined dentures. New single-jaw or full dentures were fabricated within seven to ten days. The goal was to support the patients’ general rehabilitation and to provide the masticatory preconditions necessary for protein and fiber food comminution. The aim was further to avoid or improve sarcopenia and to protect intestinal conditions. All patients were informed through verbal instruction and received written advice on how to use, adopt, and handle the reconditioned or new dentures.

### 2.3. Oral and Dental Outcome Variables and Outcome Measures

Oral and dental examinations of in-hospital patients were based on their dental files and dental status, which included the detection of caries; missing, decayed, or substituted teeth; the number and kind of restored dental units; and soft tissue inflammation and denture function. These variables defined their basic dental status [18]. Additional findings were gathered through dental anamnesis, oral examination, and interviews based on the Oral Health Impact Profile in the German language (OHIP-G5) [19,20,21]. Due to the severe health condition and mild cognitive impairment of the majority of GIH patients, the questionnaires were filled out by the dentist or physician during the interview with the patient.

The following main dental outcome variables were evaluated and statistically analyzed: dental status; the existence of pain or complaints and intra-oral risk factors (IRFs); denture status; and the need for immediate prosthetic therapy (NIPT) [18,22,23]. The Eichner Index (EI), Eichner Class/Score [16], and modified DMFT-Mod. [17] were used to classify dental arches ranging from partial to total edentulous and to plan prosthodontic treatment. The EI is based on occlusal contacts between natural teeth in the premolar and molar regions (with 2 supporting zones on each side, with a total of 4 supporting zones for the entire dentition). The Eichner classification provides a standardized measure for dentition loss and is suitable for application in studies on morbidity statistics [13,16]. In the present study, the patients were examined and three sub-types of indices for masticatory function (MF) were developed based on the Eichner Index. 

EI Standard (only natural teeth) reflected the status before any prosthetic treatment; EI Mod. #1 (Eichner Index Modified #1) described existing prostheses after prosthetic treatment but before immediate in-hospital dental intervention. This index described the number of occluding zones with existing but ill-fitting, deficient or dysfunctional prostheses. EI Mod. #2 (Eichner Index Modified #2) was recognized after in-hospital IPT and counted the number of occluding support zones of well-fitting and functional dentures. The two Eichner Index modifications introduced in this study were the Eichner Index Modified #1 and Modified #2. These became necessary because a significant portion of the measures taken involved denture relining (69% of all patients with dentures) and fracture repair (14% of all patients with dentures), which did not change the number of teeth or occluding zones. Therefore, no difference between the Eichner Index Mod. #1 and #2 would have been observed in these patients. The Standard Eichner Index as well as the Eichner Index Modified #1 and Modified #2 were calculated using numerical values for each Eichner Class: A1 = 1 up to C3 = 10. The higher the numerical value was, the less chewing function the patient had [13,14,24]. This study investigated the relationship between IRFs and masticatory function improvement (MFI) by analyzing whether EI values differed between GIH patients with and without SBCA. To assess the relationship between the dentition at baseline before IPT and the achieved improvement (MFI) after IPT, the difference was correlated and defined the primary outcome. MFI was calculated through DMFT* and Eichner Class improvement, which indicated better mastication and oral health.

The DMFT index was adapted to assess the need for prosthetic care, considering the impact of artificial teeth and dentures on the mastication of GIH patients [17]. The adapted DMFT-Mod. index included all destroyed/diseased teeth (D), missing teeth or units (M), and any necessary or completed artificial fabrication (F). Different causes, such as caries, fracture, attrition and disintegration of teeth, fixed crowns, bridge units, or removable units, along with the required care, were all summarized by “F”. This modified index was based on individual teeth, with each unit representing one tooth (T). The maximum number is 28.0, indicating that all 28 units of teeth were affected [23]. The relationship between baseline dentition and achieved MFI defined the outcome and was assessed by the difference in DMFT-Mod. before and after IPT. To assess the association of intra-oral risk factors (IRFs), such as inflammation, severe bone crest atrophy (SBCA), oral pain, and mucogingival junction insufficiency (MGJI) with health condition, the parameters were analyzed using a T-test for independent samples.

### 2.4. Oral Health-Related Quality of Life (OHRQL), Treatment Satisfaction (TS), and Outcome Measures

OHRQL questionnaires provide complementary data to the measurable clinical indicators of oral disorders, such as the indexes. Due to severe diseases, reduced cognition, and considerations of time and cost-effectiveness, short versions of the OHIP questionnaires were used, and special German short forms were selected [9,19,20,21]. In this study, we used the additionally adapted and simplified German version of OHIP-G5 for GIH patients and in-hospital requirements to provide the most practical, objective, and short survey (five short questions, simple language) before and after IPT. The questions addressed the following aspects of impaired mastication: eating discomfort/impossibility, ill-fitting or defective dentures, missing teeth, sore jaw/inflamed gum, and oral pain. When there were two or more “yes” answers, geriatric doctors at the hospital referred the patient to a dentist. The assessment of OHRQL using a questionnaire provided the indication for IPT. The association between patients’ treatment satisfaction (TS) and both the presence of at least one intra-oral risk factor and the individual IRFs (inflammation, SBCA, oral pain, MGJI) was analyzed. TS was assessed through an interview using a questionnaire focused on the objective denture function (fit, retention, balanced occlusion) and the subjective patients’ TS when the dentures were incorporated by a dentist after IPT. The outcome of IPT for GIH patients was evaluated from baseline to after treatment. Oral health-related quality of life (OHRQL) was indicated by patients’ treatment satisfaction in association with the IRFs, such as MGJI and SBCA. Treatment satisfaction, as both a subjective and objective indicator, served as the secondary outcome for OHRQL.

### 2.5. Data Collection and Statistical Analysis

All data were gathered using MS Excel (Microsoft, Redmond, WA, USA) and used for descriptive evaluation. The analyses were intended to be explorative and hypothesis-generating. Therefore, the dataset was subjected to exploratory statistical analysis, but associations and group differences were assessed using univariate testing procedures. Subsequently, the results may be unadjusted and bivariate. A significance level of *p* < 0.05 was adopted as the criterion for statistical significance. Results were annotated as follows: *p* < 0.05 (*; significant), *p* < 0.001 (**; highly significant), and *p* < 0.0001 (***; very highly significant). When statistical significance was achieved, effect sizes were computed according to the corresponding test methodology to evaluate the substantive importance of the findings. For Pearson’s correlation coefficient (*r*), applied alongside *t*-tests, Mann–Whitney tests, and Spearman’s rank correlation, the following interpretive thresholds were used: *r* < 0.1, negligible; *r* ≥ 0.1, weak; *r* ≥ 0.3, moderate; *r* ≥ 0.5, strong. For chi-squared tests, the effect size was measured using Cramer’s V, with *V* ≥ 0.30 denoting a strong association. All statistical computations were performed using SPSS Statistics version 24.0 (IBM Corp., Armonk, NY, USA).

## 3. Results

### 3.1. Descriptive Analysis After IPT and Associations with MFI

Masticatory function improvement (MFI) or Eichner Class improvement through immediate prosthetic therapy (IPT) was calculated by comparing EI scores between GIH patients (with delta = 0.20). MFI or Eichner Class improvement through immediate prosthetic therapy (IPT) was also evaluated by comparing EI scores before and after IPT. This was assessed through interviews and documentation in dental charts when the dentures were incorporated, as the dentist asked the patients about their outcomes after incorporation (Table 1).

### 3.2. Inferential Analysis After IPT and Association with OHRQL and TS

In bivariate (unadjusted) analyses, intra-oral risk factors, such as inflammation, oral pain, mucogingival junction insufficiency, and specific severe bone crest atrophy, were associated with health status as follows: The EI scores of GIH patients with and without SBCA differed significantly for EI Mod. #2 after in-hospital IPT (*n* = 47, *p* = 0.001; r = 0.38), with a medium effect size (Table 2).

To assess the influence of baseline dentition (EIs/DMFT-DMFT-Mod.) on the achieved MFI, the difference before and after IPT was analyzed. A significant positive association was found (*p* < 0.0001, *r* = 0.51), indicating a strong effect size of IPT on MFI, which was based on the comparison between EI Mod.#1 and EI Mod.#2 (Table 3).

Elderly GIH patients over 60 years with a need for prosthetic treatment (NPT) exhibited high rates of intra-oral risk factors (IRFs) such as inflammation (75%) and pain (53%) due to advanced age and high NPT. GIH patients also showed MGJI insufficiency (5%), which may be attributed to weight loss, SBCA, and high NPT. In correlation with the number of diagnoses, severe bone crest atrophy and oral pain were not significant results or reasons for this health condition. GIH patients with inflammation had significantly more diagnoses than GIH patients without inflammation. Therefore, inflammation appeared to be an independent risk factor for health status, or conversely, health status seemed to be influenced by it. In contrast, GIH patients with MGJI had significantly fewer diagnoses than GIH patients without MGJI. Consequently, MGJI did not appear to impact health status, as measured by the number of diagnoses, because it is primarily a localized and structural issue rather than a reactive oral impairment (Table S1). SBCA, but not weight loss, was significantly correlated with MFI and EI. MFI before and after in-hospital IPT was strongly affected by SBCA. GIH patients with SBCA benefited most from IPT because they suffered from worse starting conditions. GIH patients with SBCA had significantly higher EIs, indicating worse dentition, predominantly classified as C3 rather than C1. They also had significantly lower EI Mod. #2 scores, reflecting better dentition after IPT. A total of 46 out of the 47 patients with SBCA had the best possible dentition (A1) after IPT. In contrast, fewer GIH patients without SBCA (only 24 out of 34 patients) had the best possible dentition (A1) after IPT (Table 4).

The associations between baseline status and achieved MFI for each IRF in GIH patients were significant, independent of indication and inflammation, but dependent on the absence of oral pain and MGJI and the presence of SBCA.

The patients’ satisfaction after IPT was assessed through objective and subjective functional improvement. The significant association between baseline status and achieved improvement indicates that the worse the MF was before in-hospital IPT (Eichner Index Modified #1), the higher the achieved MFI was through IPT (Δ EI Mod. #1 – EI Mod. #2). This result is highly clinically relevant. Regarding the association per risk factor, it became apparent that the correlation with MFI was significant and strongly relevant, especially for GIH patients without inflammation, without oral pain, and with SBCA, and for patients without MGJI. These patients seemed to benefit most from IPT because they had poorly fitting dentures before and were in a better condition to respond to IPT. In contrast, the chance of MFI through IPT was higher in cases of SBCA (Table 5). The significant correlation of the IRF “SBCA” to patient satisfaction after IPT confirmed the need for dental care. Among all patients who received IPT in the hospital (n = 81), 78% showed treatment satisfaction with regard to objective functional improvement. There was a significant association between OHRQL, assessed by patients’ treatment satisfaction (TS) after IPT, and SBCA (*p* = 0.003). The effect size (*v* = 0.35) was interpreted as a strong association (Table 5).

## 4. Discussion

Previous research stated that the prevalence of dental morbidity is cumulative and progressive. The risk of tooth loss increases with the process of aging [25], although a decline in its prevalence and incidence has been found in multiple studies [26]. A later study found that aging, dementia, and oral health were correlated [27]. The mean number of missing teeth was significantly correlated with age, among other factors [28]. The concept of morbidity compression was introduced 30 years ago, describing the shift in disease burden to higher ages while increasing the number of healthy years of life beforehand. The data showed an ongoing significant decrease in the prevalence of tooth loss and edentulism between DMS 5 [29] and DMS 6 [30,31]. The epidemiology of edentulism generally shows a substantial global burden caused by demographic transitions and different development stages. There are significant differences between countries in terms of national income, financial support from healthcare insurance, healthcare capacity, and treatment outcomes. Scandinavian countries [11] have the smallest number of edentulous elderly individuals with the most remaining teeth [32]. The data differs in many other countries, and often the situation is worse [33,34]. In Germany, it was found that older adults retained more teeth. Accordingly, future prosthetic treatment demand is expected to shift from complete to partial dentures [31]. In the subpopulation of 75–100-year-old elderly patients requiring institutional care, which is partially comparable to this study cohort, edentulism was found in 32.8% and the DMFT was 21.6 [29]. An epidemiological population study in Germany revealed a high prevalence of edentulism and a high need for complete dentures (CDs) among75–79-year-olds. The risk of CDs increased with smoking, alcohol abuse, low education level, low household income, and age, but not with the number of diseases [35]. This association was recently confirmed for other countries [36,37].

The state of the art in methods of masticatory function assessment can be summarized. The most objective tests that evaluate mastication are masticatory ability (MA) [13], masticatory performance (MP) [14,38], and masticatory efficiency (ME) [15,39], which were used in laboratories. However, these methods require significant time, high costs, and specialized equipment and instruments [12,23]. Therefore, these methods are not appropriate for bedside assessment of GIH patients by dental practitioners during daily hospital routines. Subjective measures are based on self-perception and also depend on physical and mental health status and cognition. Therefore, OHRQL is not directly and completely detectable but assessable through questionnaires [7,8,9], which need to be shortened. Patients’ treatment satisfaction (TS) is part of OHRQL and also depends on both subjective and objective criteria. This study focused on affordable and objective dental measures of mastication. The assessment of MF through index calculations was based on dental status. This is the most adequate method for GIH patients because the information used is obtained from dental files and dental status [18]. DMFT values and the number of missing teeth were significantly correlated with age [29,36]. Eichner Classes and scores provided information on treatment needs and the outcome quality of dentures for dependent and impaired elderly individuals [32]. The immediate prosthetic treatment in this study protocol resulted in objective improvements in MF, with high objective and subjective treatment satisfaction. The association between MF and the Eichner Index has been comprehensively proven through different statistically significant results [13]. There is an emphasized need for an objective and feasible assessment of mastication because the outcomes may be imprecise or biased due to enrollment criteria or study design [40]. Recent research on frail GIH patients of similar age to this study population (mean age of 82.3 y) found that 75.8% had lost all their posterior teeth, corresponding to Eichner Class C [41].

In summary, all kinds of prosthetic treatment led to MFI. The significant association between baseline status and achieved improvement indicates that the worse the MF was before in-hospital IPT, the higher the achieved MFI through IPT. This result is highly clinically relevant.

Regarding the association of treatment success with single intra-oral risk factors, it is clear that the association with MFI was significant and strongly relevant, especially for GIH patients without inflammation, without oral pain, and with SBCA, and for patients without MGJI. The reason is that these patients seemed to benefit the most from IPT because they had no acute crisis in the oral cavity and better preconditions to successfully respond to IPT. In contrast, the likelihood of achieving MFI through IPT was higher in patients with SBCA because of the lack of denture retention and dysfunctional mastication beforehand [18].

The paradox of better subjective oral health in old age [42] refers to the phenomenon of objectively worse dental and oral status, reduced masticatory functions, and resulting impaired nutrition. There are many reasons for this, and it can lead to a “positive perception shift” and more satisfaction with existing conditions. Therefore, objective assessment of masticatory functions using index calculations is required and could be performed by dentists.

One description of oral health-related quality of life included simultaneously objective and subjective parameters (masticatory ability) [40], “as well as psycho-social, mental and well-being indicators” [43]. Objective examinations such as MP and ME confirmed the relationship between OHRQL and the occlusion of remaining teeth or the main occluding areas [44]. Another definition states that “OHRQL is a multidimensional construct that reflects people’s comfort when eating, sleeping, and engaging in social interaction and their self-esteem” [45]. In contrast, this study focused on a non-holistic but age- and dental morbidity-centered approach. Measures and treatment options were derived for and by dentists, with further impact on dental research and dental education.

Treatment outcomes following complete denture incorporation were measured objectively through masticatory performance and subjectively using OHRQL questionnaires, with both showing significant improvement after treatment [46]. Quality of life and oral health are considered components of overall quality of life for older people, with tooth loss playing a significant role despite other factors [47]. This study cohort exhibited significantly high morbidity, treatment needs, and acute dependency on rehabilitation at the hospital (Table S1). This was comparable to elderly individuals receiving home healthcare services, with a high prevalence of edentulism, non-functional or poorly fitting dentures, and therefore, a high need for prosthetic treatment, as reported in a recent review in 2022 [4]. Regarding general morbidity, it was found that our study population was similar to elderly patients hospitalized after stroke [48].

Further comparisons revealed that the need for denture treatment had the strongest association with OHRQL [49]. High general morbidity, functional impairment, and corresponding EI scores were also observed in patients with deficient dentures [41]. In contrast, one study found different patterns of prosthetic treatment needs and oral health status among functionally impaired and dependent elderly people. The reason behind this is that high-quality, expensive treatments, such as partial dentures with tooth or implant retention, are covered by the public health system in a few countries and are correlated with good functional denture status. Without tooth- or implant-supported dentures, treatment needs were higher, but only 15% of the study cohort had complete prostheses [32].

This study highlighted a significant association between SBCA and patients’ treatment satisfaction after IPT. This indicates the need for lifelong, early, and continuous dental care provided by general dentists, which can prevent caries, periodontitis, tooth loss, and subsequently poor dental and general health. In particular, this emphasizes the need for dental care for GIH patients and IPT in every hospital and nursing home. When mastication improved, it was reflected and perceived as enhanced OHRQL and activities of daily life (ADL) [18], which can provide psychological, mental and psycho-social benefits. Therefore, MFI in the elderly may be more beneficial than nutritional supplementation or medication (Table S2).

The limitations of our study include the small sample size, with later reductions in participants due to exclusion and dropout, which led to reduced informational value but great clinical significance. Possible confounding can be expected due to the heterogeneous and multimorbid nature of the GIH sample. Therefore, the results of this study are hypothetical and represent only associations. Using a single-center design and convenience sampling may introduce bias in the outcome and further reduce the generalizability of the results. The strength of our study was the use of the established and simple dental indices and scores, such as the EI and DMFT* index. The associations between intra-oral status, IPT, and OHRQL were analyzed as bivariate associations. Despite the limitations, the findings may provide new data and understanding for decision-makers in policy and healthcare systems. Routine dental rehabilitation methods may improve treatment outcomes due to their beneficial impact on general rehabilitation, low costs and easily accessible prosthetic treatments. Therefore, this study cohort may provide real-world insights into geriatric-in-hospital patients who require immediate prosthetic treatment following an interdisciplinary approach.

## 5. Conclusions

Severe bone crest atrophy appeared to be associated with masticatory function (MF) and immediate prosthetic treatment (IPT). The resulting MF improvement was the most appropriate measure for objective mastication assessment. As mastication is recognized as an important part of activities of daily life and treatment satisfaction, the results indicate oral health-related quality of life (OHRQL), especially for geriatric in-hospital patients. The benefits of in-hospital IPT were confirmed, consistent with prior clinical experience. This study provides clinical and hypothetical statistical evidence that immediate prosthetic treatment helps to improve OHRQL in GIH patients.

## Figures and Tables

**Table 1:** MFI achievement through IPT: numeric class improvement in GIH patients.

	GIH Patients (*n* = 81)			
Eichner Index (Standard)	Δ ^b^	Mod. EI #1	Δ ^c^	Mod. EI #2
Average value ^a^				
8.57	6.85	1.72	0.20	1.52

^a^ For calculations, the EIs were coded as follows: 1 = A1/a1; 2 = A2/a2; 3 = A3/a3; 4 = B1/b1; 5 = B2/b2; 6 = B3/b3; 7 = B4/b4; 8 = C1/c1; 9 = C2/c2; 10 = C3/c3. For interpretation, these values have to be rescaled. ^b^ Δ = Numeric class improvement between Eichner Index and Eichner Index Modified #1. ^c^ Δ = Numeric class improvement between Eichner Index Modified #1 and Eichner Index Modified #2.

**Table 2:** Weight loss and SBCA. Mann–Whitney tests: differences in EI scores between patients with and without weight loss and SBCA.

		Eichner Index	EI Mod. #1	EI Mod. #2
Disease	Affected	2-Sided Significance *p* (Correlation Coefficient *r*)		
Weight loss	Yes (*n* = 31)	0.799	0.351	0.144
	No (*n* = 49/50)			
SBCA	Yes (*n* = 47)	0.000 (0.46) ***	0.137	0.001 (0.38) **
	No (*n* = 33/34)			

Results were annotated as follows: *p* < 0.001 (**; highly significant), and *p* < 0.0001 (***; very highly significant).

**Table 3:** Masticatory function improvement (MFI). Association between baseline situation and achieved MFI. Scheme rank association: Association between EIs/DMFT-Mod. And MFI (difference between EI Mod. #1 and EI Mod. #2).

	Δ EI Mod. #1–EI Mod. #2
	2-Sided Significance *p* (Correlation Coefficient *r*)
EI Standard	0.564
EI Mod. #1	0.000 (0.51) ***
DMFT-Mod.	0.087

Results were annotated as follows: *p* < 0.0001 (***; very highly significant).

**Table 4:** Association between baseline and achieved MFI for each IRF. Scheme rank correlation: association between EI Mod. #1 and MFI. (Δ EI Mod.#11.–EI Mod. #2) for all patients and for each IRF.

Disease	Affected	2-Sided Significance *p*	Correlation Coefficient *r*
All patients	(*n* = 80)	0.000 ***	0.52
Inflammation	Yes (*n* = 60)	0.003 **	0.37
	No (*n* = 20)	0.000 ***	0.84
Oral pain	Yes (*n* = 42)	0.079	
	No (*n* = 37)	0.000 ***	0.70
SBCA	Yes (*n* = 47)	0.000 ***	0.86
	No (*n* = 33)	0.073	0.32
MGJI	Yes (*n* = 4)	n too low	
	No (*n* = 76)	0.000 ***	0.54

“Yes” means presence of muco-gingival joint insufficiency or reduced function and size; “No” means absence of MGJI or healthy, functional, adequately sized and stable vestibular jaw tissues, including each lower and upper frenulum of the lip or cheek. Results were annotated as follows: *p* < 0.001 (**; highly significant), and *p* < 0.0001 (***; very highly significant).

**Table 5:** Bivariate associations of intra-oral risk factors with treatment satisfaction (TS) after IPT. Pearson’s chi-squared tests: correlation between patients’ TS and IRF.

	Patients’ TS
	2-Sided Exact Significance *p* (Cramer-V *v*)
All IRF	1.000
Inflammation	0.720
SBCA	0.003 ** (0.35)
Oral Pain	0.357
MGJI	0.102

Results were annotated as follows: *p* < 0.001 (**; highly significant).

## Data Availability

The data presented in this publication are available on request from the corresponding author. The data are not publicly available due to privacy restrictions.

## Supplementary Materials

The following supporting information can be downloaded at https://www.mdpi.com/article/10.3390/dj14050268/s1: Table S1: Baseline demographic data and health status of GIH patients; Table S2: Comparison between single GIH cohort (Coburg) and total federated state rehab population: demographic, geriatric, and mental measures.

## Abbreviations

The following abbreviations are used in the manuscript:

ADL	Activity of Daily Life
CD	Complete Denture
EI	Eichner Index
DMFT	Decayed-Missing-Filled Index per Tooth
DMFT-Mod.	Destroyed/Diseased-Missing-Teeth/Unit-Fabrication Index per Tooth/Unit, Mod. = modified
GIH	Geriatric In Hospital
IPT	Immediate Prosthetic Treatment
IRF	Intra-oral Risk Factor
MCI	Mild Cognitive Impairment
MGJI	Muco-Gingival Joint Insufficiency
MA	Masticatory Ability
ME	Masticatory Efficiency
MP	Masticatory Performance
MFI	Masticatory Function Improvement
Mod.	Modified
NIPT	Need of Immediate Prosthetic Treatment
OHIP	Oral Health Impact Profile
OHRQL	Oral Health-Related Quality of Life
SBCA	Severe Bone Crest Atrophy
TS	Treatment Satisfaction
