# Supplementary material for: Masticatory Function and Oral Health-Related Quality of Life After Immediate Prosthetic Therapy for Geriatric In-Hospital Patients: A Retrospective Cohort Study
Source: Dent J (Basel). 2026 May 4;14(5):268. doi: 10.3390/dj14050268 (PMC13205564; doi:10.3390/dj14050268)
Supplement: Supplementary file 1 [file dentistry-14-00268-s001.zip › dentistry-4123785-supplementary.pdf]

Table S1. Baseline demographic data and health status of GIH patients.

Demographic and Health Status	GIH Patients (n = 81)
Age	81.9
Gender	
Male [%]	38
Female [%]	62
(Oral) health status	
No. of medical diagnoses [M ± SD]	13.86 ± 4.73
Need for general dental care [%]	41
Need for removable prosthetic therapy [%]	74
Need for fixed prosthetic therapy [%]	5
Orofacial/neurological risk factors [%]	
CNS disorders	44
MCI	31
Orofacial disorders	10
Intra-oral risk factors [%]	
Inflammation	75
SBCA	58
Oral Pain	53
MGJ	5
	GIH (n = 81)
Average DMF*-T	25.58

Note: CNS = central nervous system. SBCA = severe bone crest atrophy. MGJ = mucogingival-junction insufficiency. MCI = Mild Cognitive Impairment. DMF*-T = adapted version of the measure of the DMF-T index used to measure dental morbidity.

Table S2. Comparison between single GIH cohort (Coburg) and total federate state rehab population: demographic, geriatric, and mental measures.

	Coburg GIH Rehab Department	Total Number of Rehab Departments in Bavaria 2015–2017 Without Patients from Coburg
Number of cases	61	107,347
Age (years)	82.02	81.53
Proportion of women (%)	69.3	66.79
Length of stay (days)	27.93	21.79
Barthel index at admission	35.67	46.54
Barthel index at discharge	58.67	68.67
MMSE	22.39	24.42
TUG at admission (%) [a]	33.33–35.29–31.37	55.74–29.28–14.98
TUG at discharge (%) [a]	84.09–11.36–4.55	79.77–15.18–5.05
CCI	4.46	4.69

Note: This data does not originate from this study but from the GiB-DAT databank and serves as a comparison to our results. Only 61 patients out of our study group (n = 81) could be calculated due to missing data. MMSE = mini mental state examination. TUG = timed up and go test. CCI = Charlson Comorbidity Index. [a] Independently ambulatory, ambulatory with assistance, not ambulatory.
